# Response to: Surgical approach for totally implantable venous access port: a full strategy to avoid the percutaneous approach complications

**DOI:** 10.1007/s00423-021-02159-z

**Published:** 2021-04-16

**Authors:** Ulla Klaiber, Phillip Knebel

**Affiliations:** Department of General, Visceral and Transplantation Surgery, University of Heidelberg, Im Neuenheimer Feld 420, 69120 Heidelberg, Germany

We thank our Italian colleagues for their interest in our work [1], and we appreciate their statement in favor of surgical cut-down of the coracobrachial or external jugular vein as an alternative in case open cut-down of the cephalic vein fails. We share their opinion that this is a reasonable approach as it offers the chance to prevent puncture-associated complications such as pneumo- and hemothorax effectively. We congratulate Professor Di Carlo and co-workers for their outstanding work on surgical techniques for implantation of totally implantable venous access ports (TIVAP) [2, 3]. To our knowledge, outcomes of surgical cut-down of the coracobrachial or external jugular vein for TIVAP implantation have not been investigated within a randomized trial design yet. Therefore, high-quality trials on this topic are needed to gain better evidence as a basis for practice recommendations and guidelines. The conclusion drawn from our results [1] is based on meta-analyzed data from randomized controlled trials in which venous puncture was used as second- or third-line strategy. Considering that complication rates were low in our study even when a percutaneous technique was used, our recommendations were justified and evidence based.
